# A Model of *Salmonella* Colitis with Features of Diarrhea in *SLC11A1* Wild-Type Mice

**DOI:** 10.1371/journal.pone.0001603

**Published:** 2008-02-13

**Authors:** Heungjeong Woo, Sharon Okamoto, Donald Guiney, John S. Gunn, Joshua Fierer

**Affiliations:** 1 Department of Infectious Diseases, Veterans Affairs (VA) Healthcare, San Diego, California, United States of America; 2 Division of Infectious Diseases, Department of Medicine, University of California San Diego School of Medicine, San Diego, California, United States of America; 3 Center for Microbial Interface Biology, Department of Molecular Virology, Immunology, and Medical Genetics, The Ohio State University, Columbus, Ohio, United States of America; Centre for DNA Fingerprinting and Diagnostics, India

## Abstract

**Background:**

Mice do not get diarrhea when orally infected with *S. enterica*, but pre-treatment with oral aminoglycosides makes them susceptible to *Salmonella* colitis. However, genetically susceptible ItyS mice (Nramp1^G169D^ allele) die from systemic infection before they develop diarrhea, so a new model is needed to study the pathogenesis of diarrhea. We pretreated ItyR mice (Nramp1^G169^) with oral kanamycin prior to infecting them with virulent *S*. Typhimurium strain 14028s in order to study *Salmonella-*induced diarrhea. We used both a visual scoring system and the measurement of fecal water content to measure diarrhea. BALB/c.D2^Nramp1^ congenic started losing weight 5 days post-infection and they began to die from colitis 10–14 days after infection. A SPI-1 (*invA*) mutant caused cecal, but not colonic inflammation and did not cause diarrhea. A *phoP-* mutant did not cause manifestations of diarrhea in either normal or NADPH-deficient (gp91*^phox^*) mice. However, strain 14028s caused severe colitis and diarrhea in gp91^phox^-deficient mice on an ItyR background. *pmr* A and F mutants, which are less virulent in orally infected BALB/c mice, were fully virulent in this model of colitis.

**Conclusions:**

*S. enterica* must be able to invade the colonic epithelium and to persist in the colon in order to cause colitis with manifestations of diarrhea. The NADPH oxidase is not required for diarrhea in *Salmonella* colitis. Furthermore, a *Salmonella phoP* mutant can be cleared from the colon by non-oxidative host defenses.

## Introduction

*Salmonella* gastroenteritis is a global health problem, even in industrialized nations. The Center for Disease Control (CDC) estimates that there are nearly 1.4 million foodborne *Salmonella* infections annually in the USA [1]. Even though most *Salmonella* intestinal infections are self-limited, the illness usually lasts 3–5 days and is estimated to cost $1.4 billon/year in lost wages, expense of recalls, and medical costs [2]. Furthermore, there has been a steady increase in the prevalence of antibiotic resistant *S*. *enterica* serovar Typhimurium (hereafter called *S.* Typhimurium) [3], and a recent study from CDC found that 20% of victims in food borne epidemics caused by antibiotic resistant *S. enterica* required hospitalization, emphasizing that even gastroenteritis can be a serious disease [4].

*S. enterica* are invasive enteric pathogens, but the pathogenesis of diarrhea in *Salmonella* gastroenteritis is poorly understood. Many investigators have focused on mechanisms of cell invasion using cultured epithelial cells. This led to the discovery of a type 3 secretion system (TTSS) that is encoded by a Salmonella pathogenicity island (SPI-1). The TTSS mediates epithelial cell invasion by injecting bacterial products into host cells that rearrange the host cytoskeleton, causing localized membrane ruffling where the bacteria enter [5],[6],[7]. Uptake of *Salmonella* also triggers a complex transcriptional response in epithelial cells that results in expression of many NF-κB regulated genes. Since most of those genes encode pro-inflammatory cytokines and chemokines this response has the potential to initiate inflammation, and inflammation is a prominent feature of *Salmonella* gastroenteritis. The induction and basal secretion of chemokines by polarized epithelial cells occurs rapidly [8], [9]. Many of the *Salmonella* -induced changes initially observed in cultured cell lines have been confirmed in vivo in bovines and in human intestinal xenografts in SCID mice [10],[11]. However, it is likely that *Salmonella* induce additional epithelial responses that are not apparent in vitro because cell lines do not recapitulate all of the properties of the native intestinal epithelium.

Mice do not develop diarrhea when orally infected with *S. enterica*, which has greatly hampered the study of the pathogenesis of intestinal infection. However, a mouse model of *Salmonella* colitis in streptomycin-pretreated mice was developed more than fifty years ago by Bohnhoff [12]. Over the subsequent 20 years several groups used this model to show that oral streptomycin makes mice 100,000 fold more susceptible to oral *Salmonella* infection, and that alterations in the normal intestinal microflora are responsible for their increased susceptibility. Indeed, germ free mice are also susceptible to *Salmonella* colitis [13]. This model languished until Hardt's group in Switzerland recently revived it. They confirmed that the streptomycin-treated BALB/c and C57BL/6 (B6) mice get an acute colitis when infected orally with *S.* Typhimurium [14]. Most importantly, they showed that SPI-1 mutants of *S*. Typhimurium (*invJ* and *invG* ) did not produce colitis, though they still could infect the cecum as well as wild type (WT) *Salmonella,* and they were also invasive as measured by CFU in the mesenteric lymph nodes [14]. Hapfelmeier *et al* [15] then showed that SPI-1 effector molecules SipA, SopE, and SopE2 were involved in producing the pathology of *Salmonella* colitis. Thus, they established the physiological relevance of the model.

Nearly all reported experiments on *Salmonella* colitis in mice have been done with *Nramp1^G169D^* mutant mice (ItyS) that are so susceptible to systemic *Salmonella* infections that they die 4–5 days after oral infection. Perhaps because of that, most studies of *Salmonella* colitis in mice have focused on cecal inflammation [15],[14],[16]. Although investigators refer to it as a model of *Salmonella* colitis, inflammation and gross pathology are focused on the cecum with some inflammation in the contiguous colon. *Nramp1* is encoded by a gene on chromosome 1 that is now called *Slc11a1* [17]. Normal animals do not have mutations in this gene. We began to study *Salmonella* colitis in ItyR (Nramp^G169^) mice. This paper reports our results with this model of colitis, a model of pan colitis with features of diarrhea.

## Results

**Figure 1:**
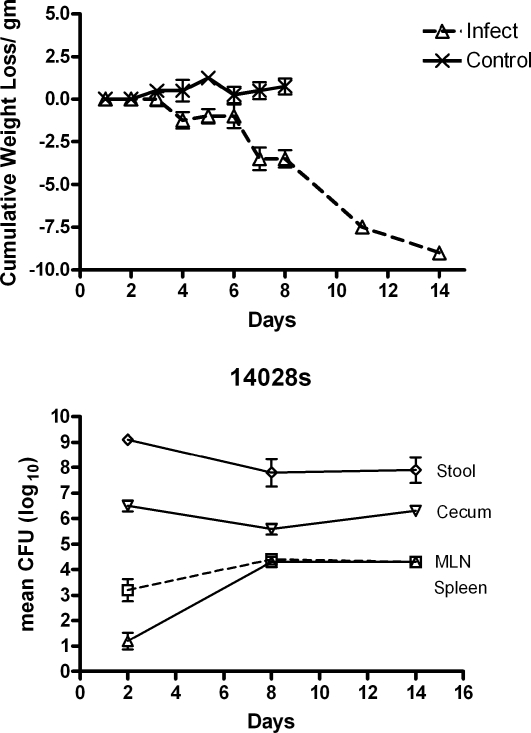
Time course of infection in BALB/c.D2^Nramp1^ congenic mice with Salmonella colitis. A. Cumulative mean weight loss of mice that were infected with *S.* Typhimurium strain 14028s after pretreatment with oral kanamycin, compared to controls that received only oral kanamycin. (The controls were not followed beyond 8 days.) B. Numbers of *Salmonella* (geometric mean CFU±SEM) recovered from feces, ceca, mesenteric nodes, and spleens at indicated time points after infection. There were 5 mice at each time point except for day 14 when there were only 3 mice.

**Figure 2:**
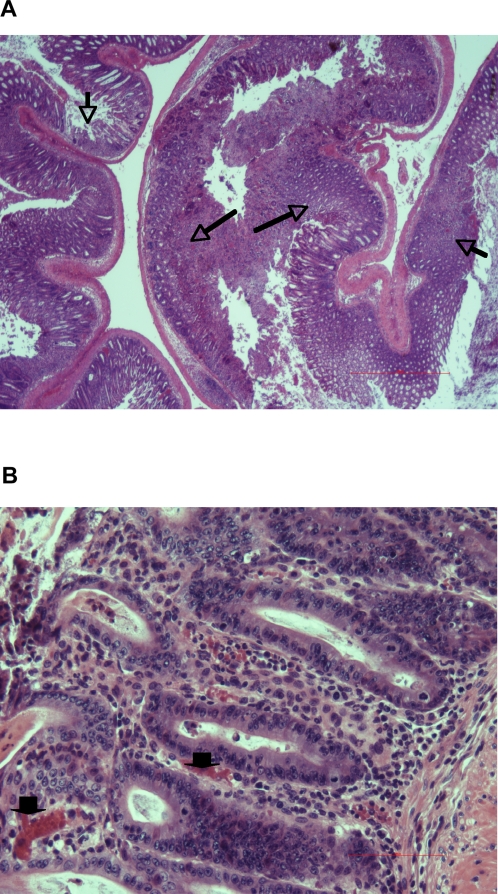
WT *Salmonella* causes extensive colitis in BALB/c.D2 mice. A. H&E stained section from a representative mouse showing two adjacent turns of the colon, 9 days after infection, at low magnification (40X). Note the extensive inflammation throughout the colon with many mucosal ulcers (arrows), and exudation of inflammatory cells and sloughed epithelium from opposing sides of a segment of colon merging in the lumen. B. Higher power (200X) view of the colonic mucosa from the same mouse. Note the infiltration of inflammatory cells (PMN and mononuclear cells), mucosal hemorrhage (Arrow heads), and mucosal ulceration. There was no sub-mucosal edema in this segment but there was extensive edema in other areas of the section (see B).

We first compared the natural history of oral infection with *S.* Typhimurium in kanamycin-treated BALB/c and BALB/c.D2^Nramp1^ mice. As expected, all the BALB/c mice were dead by 5 days after infection and they had 10^6^–10^7^ CFU/spleen at the time of death. We expected the BALB/c.D2^Nramp1^ congenic mice to survive and to spontaneously clear the infection, but instead they started to lose weight by day 5–6 after infection (Figure 1A) and they had visible evidence of diarrhea as manifested by fecal soiling of their peri-anal fur. By day 14 post infection the mice had lost about 30% of their body weight. We repeated the experiment to do quantitative microbiology on the tissues (Figure 1B). Between days 2 and 8 after infection there was a 1 log increase in the number of *Salmonella* in the mesenteric nodes and a 3 log increase in the number of bacteria in the spleen, but over the next five days there was no further increase in numbers of Salmonella in those organs, even though about half the remaining mice in this experiment died between days 8 and 14 after infection. Numbers of gentamicin-resistant (intracellular) *Salmonella* in their ceca remained fairly constant throughout this time period. Even though BALB/c.D2^Nramp1^ congenic mice could not clear the intestinal infection, colony counts of *Salmonella* in the spleen were well below the numbers that were found in genetically susceptible BALB/c mice at the time of death. On day 2 after infection inflammation was limited to the cecum (not shown), but by 8 days after infection in the pathologic process had progressed to involve the entire colon with many mucosal ulcers scattered throughout (Figure 2), and the mice had watery feces in their distal colon, a feature of diarrhea.

**Figure 3:**
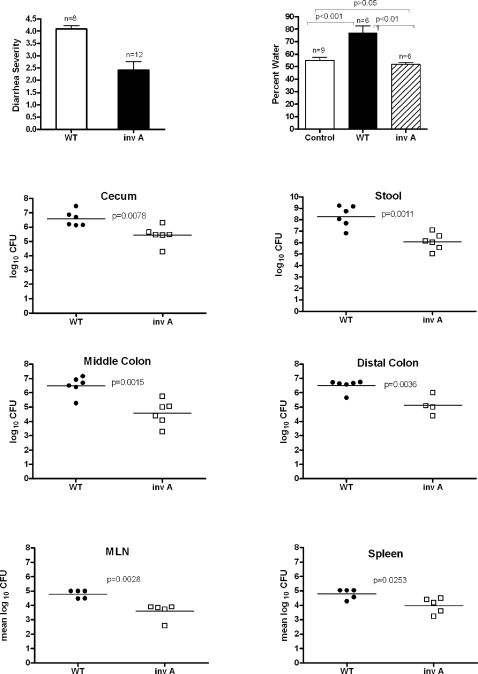
A SPI-1 (*invA*) mutant of 14028s does not cause diarrhea despite being able to invade the colons of BALB/c.D2^Nramp1^ congenic mice. A. Diarrhea scores (mean±1SEM) for mice infected with 14028s (WT) or the *invA* mutant, 8 or 9 days after infection. The numbers of mice in each group are shown above the bars. The difference in means (Mann Whitney U test) was significant (P<0.05). B. Mean water content of feces from uninfected (control), 14028a-infected (WT), and *invA*-infected mice 8 days after infection. There was no significant difference between the % of water in feces from *invA*-infected and from control mice. The % of water in feces from WT–infected mice was significantly greater than the % in the other two groups. C. CFU from feces and three segments of the colon 8 days after infection with either WT or *invA*- *Salmonella*. Each symbol represents one mouse and the horizontal bars show the geometric means. P values were calculated using the Mann-Whitney U test.

**Figure 4:**
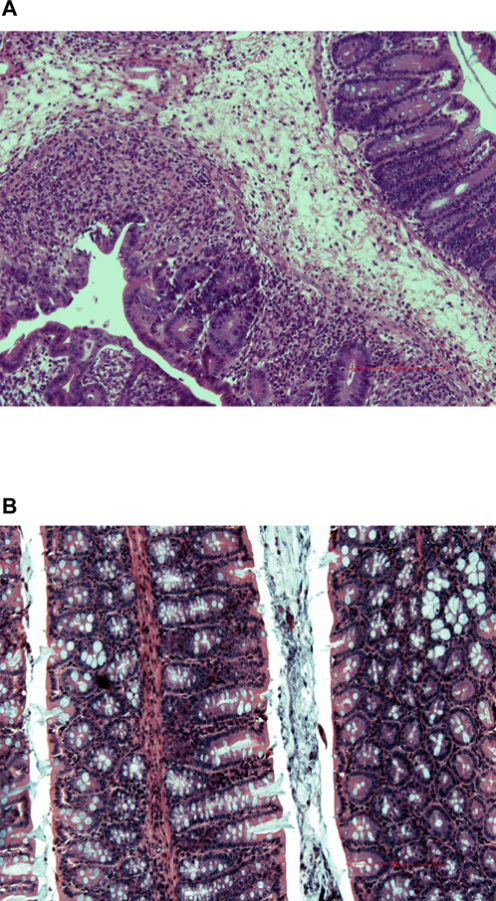
*invA* causes inflammation in the cecum but not the distal colon. A. H&E stained section (100X) of the cecum from a representative mouse 9 days after infection with *invA*-. Note the extensive sub-mucosal edema (asterisk), inflammatory infiltrate, and an area of mucosal ulceration. B. H&E stained section of 3 turns of the colon from the same mouse, showing normal mucosal architecture with essentially no inflammation and normal numbers of mucus secreting cells.

To investigate the role of SPI-1 in the pathogenesis of colitis and diarrhea we infected BALB/c.D2^Nramp1^ mice with an *invA* mutant of 14028s, which has no functional SPI-1 secretion system and so cannot invade epithelial cells in vitro [18]. Mice infected with this mutant lost less than 10% of their body weight and by day 8 after infection they had a mean diarrhea score of only 2, compared to a diarrhea score of >4 in mice infected with WT (Figure 3). Furthermore, the mean water content of the feces on day 8 was no greater than that of uninfected control mice, while feces from WT infected mice were nearly 80% water (Figure 3B). As Barthel et al previously reported, after 48 hours there are nearly equal numbers of SPI-1 mutants and WT *Salmonella* in the feces, cecal wall, and mesenteric nodes (data not shown). However, by day 8 after infection there were approximately 10 times more WT bacteria than *invA* mutants in ceca, and the differences were even greater in the feces, the mid and distal portions of the colon (Figure 3C). There also were more WT than *invA* organisms in the mesenteric nodes and spleen, though the differences between the groups were not as great as we found in the colon samples and they may have reflected the difference in the extent of infection in the colon. Cecal inflammation remained prominent in the *invA* group on day 8 post-infection (Figure 4A) but there was essentially no inflammation in the mid or distal colon (Figure 4).

**Figure 5:**
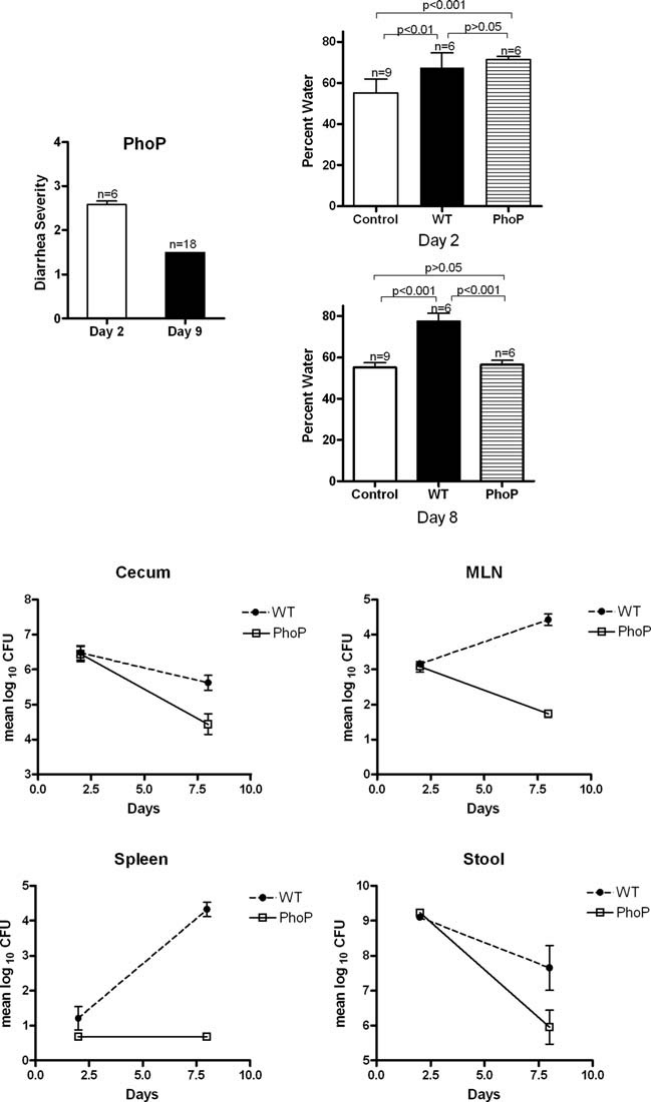
A *phoP* mutant of 14028s invades the colon, but does not persist in the colon, and does not cause colitis in BALB/c.D2^Nramp1^ congenic mice. A. Diarrhea scores for *phoP-*infected mice on days 2 and 8/9 after infection. The score is significantly higher on day 2 (P<0.05). B. Water content of the stool on those days. The water content of the stool of *phoP*-infected mice on day 2 after infection was significantly higher than that of uninfected control mice and not significantly different from the water content of mice infected with WT *Salmonella* (14028s). However, by day 8 the water content of feces from mice infected with WT had increased to nearly 80% while the water content of feces from *phoP*-infected mice had returned to normal. C. Comparison of the numbers of CFU of WT and *phoP* Salmonella on days 2 and 7 after infection. Geometric means±1SEM are shown. * =  P <0.05, ** =  <0.001. P values for the differences between means were calculated using the Mann-Whitney test. There were 6 mice/group. We did not calculate P values for spleen since there were <10 CFU of the *phoP* mutant, or for the CFU in stool on day 8 since we had only 3 stools from WT mice for culture.

**Figure 6:**
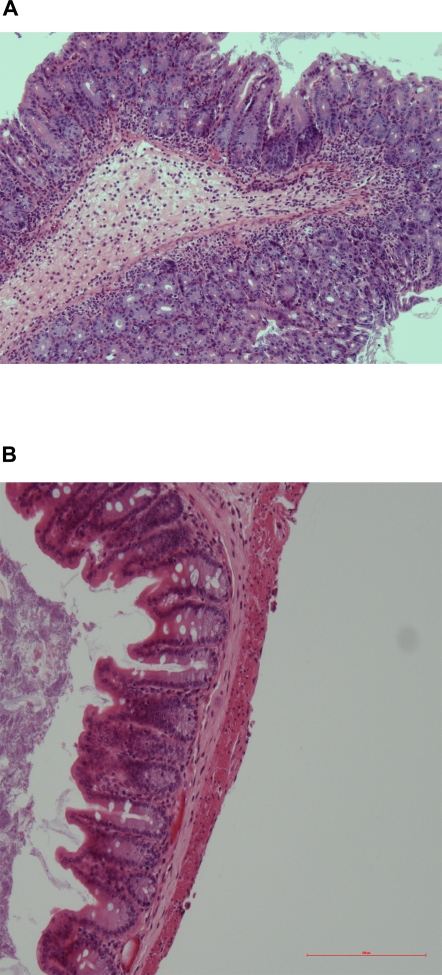
*phoP* cause transient inflammation of the cecum in BALB/c.D2^Nramp1^ congenic mice. A. H&E stained section of the cecum of a representative mouse 2 days after infection (100x). Note the edema, intense mixed cellular infiltrate in the mucosa and sub-mucosa, and the paucity of mucus secreting epithelial cells. B. A cecum from a representative mouse 9 days after infection with *phoP* (100X). The tissue is normal in appearance (100X).

We then infected BALB/c.D2^Nramp1^ mice with a *phoP* mutant [19]. On day 2 after infection *phoP*-infected mice had a mean diarrhea score of 2.5 and an increased water content of their feces, both of which were similar to results from WT infection (Figure 5A&B). However, by day 8/9 after infection the *phoP* diarrhea score had decreased to 1.5 and the water content of their colonic feces was now normal, whereas the water content of colonic feces from mice infected with WT Salmonella was ∼80%. On day 8 after infection there were significantly fewer *phoP-* than WT *Salmonella* in the stool, cecum, and mesenteric nodes (Figure 5C). The pathology in the cecum also changed between day 2 and 9 after infection: on the second day the pathology was indistinguishable from a WT infection (Figure 6A), but on day 9 the mucosa looked essentially normal (Figure 6B). The colons were not inflamed on either day (not shown). The *phoP* mutant did not infect their spleens at either time point. In other organs the *phoP* mutants were killed.

**Figure 7:**
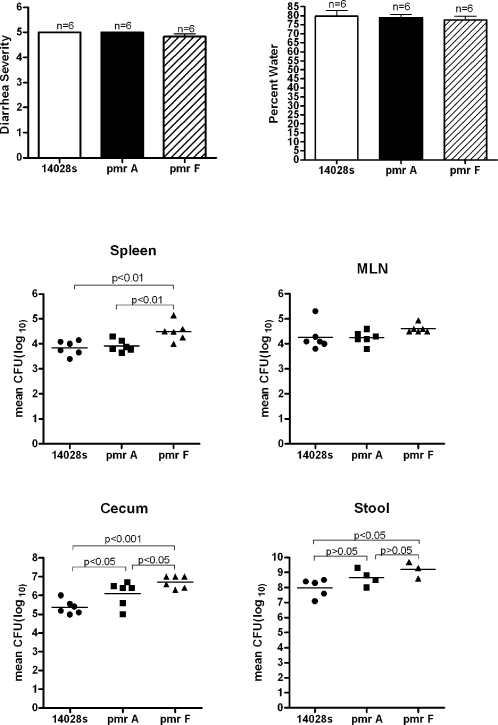
*pmrA* and *pmrF* mutants cause severe colitis. BALB/c.D2 mice were infected as in Methods and sacrificed 8 days later. There were no statistically significant differences between the diarrhea scores or the water content of feces from mice infected with WT or the isogenic *pmrA* or *pmrF* mutants. The numbers of WT and mutant *Salmonella* that we recovered from the tissues on day 8 after infection were either not significantly different or the *pmr* mutants were slightly more numerous.

Because *phoP* indirectly regulates *pmrA/B*, which regulates genes that modify the LPS and enhance the resistance of Salmonella to antimicrobial peptides such as polymyxins, we also tested *pmrA* and *pmrF* mutants in the colitis model. In contrast to the *phoP* mutant, the two *pmr* mutants were fully virulent, producing severe diarrhea (Figure 7A), multiplying in the colon, and spreading systemically (Figure 7B). The numbers of *pmrA* and *pmrF* mutants recovered from the cecum and stool were slightly greater than WT.

**Figure 8:**
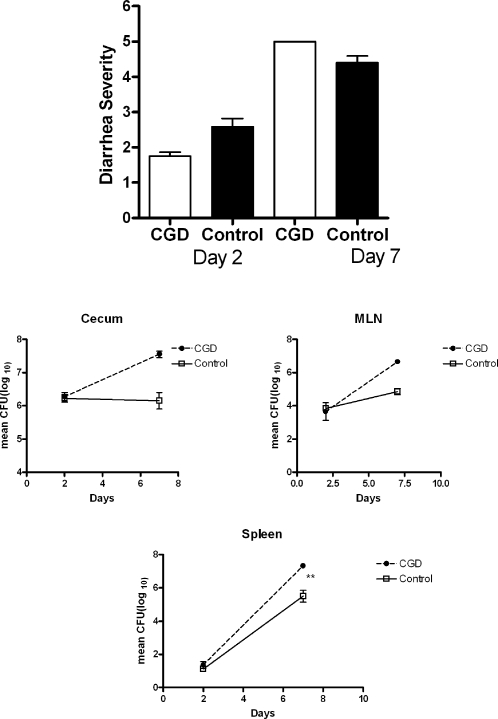
Mice with CGD are more susceptible to *Salmonella* colitis. Male mice with CGD and littermate controls (female carriers) were bred so that they carried one copy of an Nramp1^G169^ allele. Mice were infected as in Methods and sacrificed 7 days later. We infected 12 mice/group and sacrificed half of them on day 2. A. Diarrhea scores for CGD mice and littermate controls 2 and 7 days after infection. The mean diarrhea score for CGD mice was significantly higher than scores for the controls on day 2 (P<0.05), but the scores for the two strains were not significantly different on day 7, although there were only 3 surviving CGD mice on day 7. B. Geometric mean CFU±SEM for various tissues on days 2 and 7 after infection. Colony counts were similar in the two groups on day two but significantly higher in the CGD mice on day 7 after infection. * indicates a P value (Mann Whitney U test) of <0.01. On day 7 there were no solid feces in the colons of CGD mice so we could not culture their stool, and only 2/6 females had solid feces to culture.

We then turned our attention to investigating host factors that might contribute to diarrhea. *Salmonella* gastroenteritis is an inflammatory condition with large numbers of PMN in the tissues for at least the first 5 days after infection [15],[14]. Because neutrophils can make a vigorous oxidative burst and people with CGD are more susceptible to *S. enterica* bacteremia [20], we tested the role of reactive oxygen in both host defense and in mediating diarrhea. We infected mice with chronic granulomatous disease (CGD) due to a targeted mutation in gp91^phox^ on the X chromosome [21]. We crossed homozygous gp91^phox^ mutant females with transgenic TF6 males so that male offspring had CGD and female offspring were carriers. Both sexes inherited one allele of the Nramp1^G169^ transgene, which is dominant, so they were ItyR. After 48 hours both male and female mice had inflamed ceca (not shown) and there was no significant difference between their diarrhea scores or in the numbers of *Salmonella* that we recovered from their ceca (Figure 8). We terminated the experiment on day 7 because ½ of the infected males with CGD had died (none of the female carriers died). However, all the female carriers of the gp91^phox^ mutation had full blown diarrhea by day 7 (mean diarrhea score 4.5) as happens in ItyR mice, and the 3 remaining males with CGD all had diarrhea scores of 5 (Figure 8A). As expected, we recovered significantly more *Salmonella* from the male CGD mice than from the female carriers on day 7 (Figure 8B).

**Figure 9:**
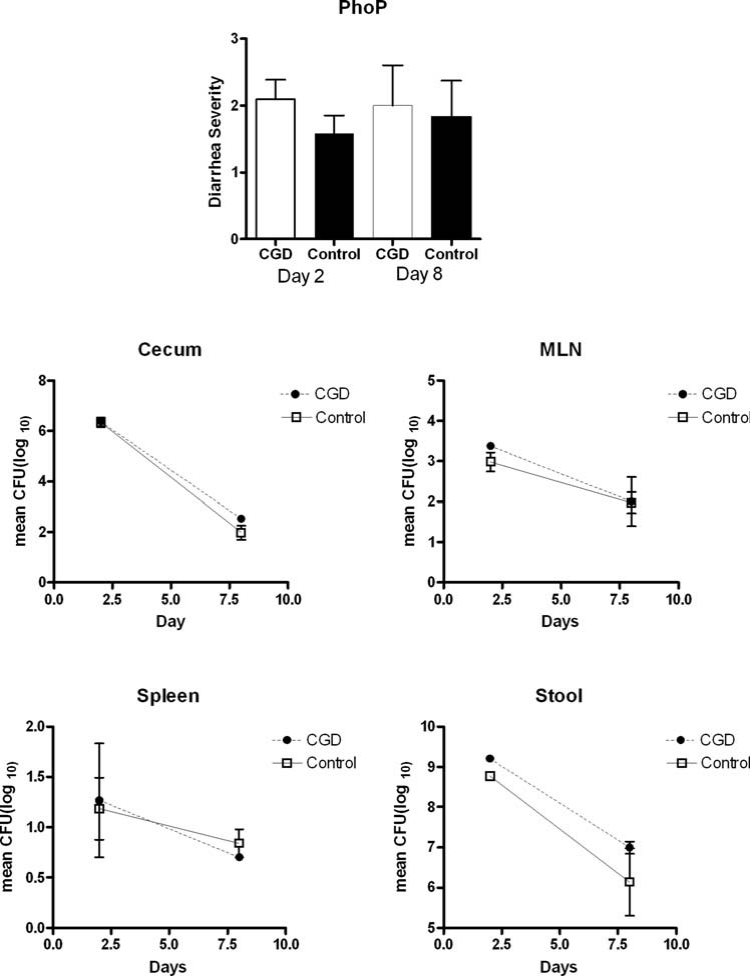
Mice with CGD are not more susceptible to oral infection with a *phoP* mutant *Salmonella*. A. Mean±SD of diarrhea scores of mice with CGD and controls (female littermates), 2 and 8 days after infection. There was no significant difference between the diarrhea scores of the two groups on either day. B. Quantitative bacteriology results for mice with CGD and control. There were no significant differences between CFU recovered from mice with CGD and littermate controls on either day. Note that by day 8 there were only ∼200 CFU recovered from the ceca of both groups, and there were <10 CFU recovered from spleen cultures. , indicating that in vivo the NADPH oxidase was not involved in the highly effective innate immune response that can resolve an oral infection with *phoP* mutants.

Since it has been reported that *phoP* mutants are more susceptible to oxidative killing in vitro [22], we asked whether *phoP* mutants would be more virulent in CGD mice, and they were not. Neither the males nor the females infected with the *phoP* mutant had diarrhea on day 8 after infection (Figure 9A). As we had seen with BALB/c.D2^Nramp1^ congenic mice, the *phoP* mutant bacteria were able to invade the intestinal wall, but by day 8 they were nearly completely eliminated from the ceca and mesenteric nodes of both CGD and control mice (Figure 9B).

## Discussion

We used a mouse model of *Salmonella* colitis in BALB/c.D2^Nramp1^ mice, which have a wild type *Nramp1* (*Slc11a1*). Because they expressed Nramp1 in their macrophages, they were better able to inhibit the growth of virulent *Salmonella* in their tissues, which allowed the mice to survive an infection with *S.* Typhimurium 14028s for at least 10 days. However, colitis progressed during that time and the mice developed more signs of diarrhea. Diarrhea in humans is often defined as an increase in the number and/or the volume of bowel movements per day, or the passage of unformed stools that assume the shape of the container. When it has been measured, the water content of diarrheal stools is greater than do solid stools [23] ,[24]. Since we could not measure the fecal output of mice directly and we could only infer the presence of unformed stools by fecal soiling of their peri-anal fur, we developed a scoring system to rate the severity of diarrhea based on the gross appearance of the colon at necropsy. A normal appearing colon containing formed feces was given a score of 1. If the cecum was shrunken and white it was scored as 2. We gave the highest score (5) to a mouse that had a shrunken cecum and a distal colon that was fluid-filled with little or no formed stool (Table 1).

**Table 1:** Scoring system for grading diarrhea.

Grade	Criteria
1	normal colon with normal formed feces in the distal colon or rectum
2	cecum that is smaller than normal with areas of white and/or gas bubbles in the lumen; the rest of the colon is normal
3	severe typhlitis (white, shrunken cecum) with involvement of the proximal colon so that it is fluid filled; formed stools in distal colon
4	Grade 3 plus fluid throughout most of the colon with soft feces in the distal colon
5	no formed feces in the entire colon and the cecum white and shrunken; colon filled with clear or bloody mucus.

Because the diarrhea score was somewhat subjective, we sought to validate it by measuring the water content of feces in the distal 3 cm of the colon, using a modification of the method of Guttman et al, who measured fecal water content from the distal colon as part of a study of the pathogenesis of diarrhea induced by *Citrobacter rodentium* [25]. For this measurement it was necessary for us to analyze the fecal material from the distal colon rather than defecated stool, since mice with diarrhea scores of 4–5 did not pass formed stools. Because the colon absorbs water from the feces [26], we cannot be certain that the water content of defecated feces and the water content of the fecal material in the distal colon would have been the same, had we been able to collect defecated feces from the infected mice. However, using uninfected mice the water content of defecated feces with that of feces taken from the distal 2 cm, or from the distal 3 cm of the colon (unpublished results, jf), and there was no significant difference. Thus, in normal mice the bulk of water is absorbed more proximally in the colon and that encourages us that our measurement of fecal water is a valid surrogate for the signs that are used clinically to define diarrhea.

We used this mouse model to begin to investigate pathogen and host factors that contribute to diarrhea due to *S. enterica* infections. *S. enterica* are a common cause of infectious diarrhea, but the mechanism of diarrhea in this infection is not well understood. Prior studies of the mouse model of *Salmonella* colitis have focused primarily on the pathogenesis of inflammation [16],[27],[28], [29], possibly because ItyS mice (such as BALB/c and C57BL/6) do not survive beyond 4 or 5 days, and they do not have obvious diarrhea before they die. Our focus was on the pathogenesis of diarrhea, although we realized that diarrhea may be secondary to the inflammatory response.

To begin to assess the role of bacterial virulence factors in *Salmonella* colitis in mice we infected them with SPI-1 (*invA*) or *phoP* mutants. SPI-1 is required for invasion of cultured epithelial cells [30] and SPI-1 mutants have been studied previously in aminoglycoside pre-treated ItyS mice. Rollenhagen and Bumann showed that SPI-1 genes are transcribed in the colon of mice with colitis [31]. Surprisingly, despite their inability to invade cultured epithelial cells, SPI-1 mutants invade the cecum as well as isogenic WT, as measured by bacteria recovered from the cecum [14]. SPI-1 mutants may be taken into the colon by mucosal dendritic cells, bypassing the epithelium [32]. Once in the submucosa where they can interact with macrophages and mesenchymal cells, inducing them to make the same panoply of pro-inflammatory molecules in a MyD88/NF-κB dependent manner [33], they cause inflammation [15],[14],[34]. There is direct visual evidence that SPI-1 mutants localize in the lamina propria but not in epithelial cells [27]. Hapfelmeier *et al* used GFP expressing *Salmonella* with mutations in either SPI-1 or SPI-2 to localize WT and mutant bacteria in the cecum, and they found that WT *Salmonella* were in both epithelial cells and the lamina propria, while SPI-2 mutants were found only in the epithelial layer. Two laboratories recently reported that the SPI-2 pathogenicity island also contributes significantly to cecal inflammation [27], [16]. Hapfelmeier *et al* reported that the residual inflammation caused by infection with a SPI-1 mutant was reduced significantly if mice had a mutation in MyD88, which abrogates most signaling through TLRs [27]. In contrast, inflammation produced by a SPI-2 mutant (expressing an intact SPI-1) was not much reduced in MyD88 mutant mice. This implies that *Salmonella* can induce colonic inflammation in two ways. SPI-1 promotes epithelial invasion, which stimulates those cells to make chemokines and pro-inflammatory cytokines [35], independent of MyD88 signaling. SPI-1 mutants do not stimulate epithelial cells but they do survive inside macrophages (SPI-2 is intact) and those cells are stimulated to make cytokines and chemokines in a MyD88-dependent fashion. Which TLR is responsible for the inflammation caused by SPI-1 mutants is not known but both TLR4 and TLR5 are expressed by infiltrating macrophages [36].

We too found that WT and a SPI-1 mutant (*invA*) invaded the cecum and caused significant inflammation, but we found that WT *Salmonella* infection spread distally to infect and inflame most of the colon by day 8/9 after infection, while the SPI-1 (*invA*) mutant remained largely confined to the cecum, as did the pathology. Most importantly, the diarrhea scores and the water content of the feces from mice infected with the *invA* mutant were significantly lower than the scores produced by WT Salmonella (Figure 3). The localization of inflammation produced by *invA* to the cecum may explain why *invA* did not cause significant diarrhea in this model of colitis. It is not clear why the cecum was more susceptible to *invA* infections than the rest of the colon. The terminal ileum enters the cecum and that part of the colon is patulous, which may allow more time for the bacteria to be in contact with the mucosa. Alternatively, there may be differences in the residual flora of the cecum and the rest of the colon, though we did not study that. *invA Salmonella* may also be taken up by M cells that overlie Peyer's patches, which are present in the cecum. Hoever, Hapfelmeier et al [27] showed that WT and SPI-1 mutants can invade the ceca of LTβR -/- mice, which have no Peyer's patches or GALT, so we cannot attribute susceptibility to the lymphoid aggregates that normally are in the mouse cecum.

We also studied a *phoP* mutant in this model. PhoP mutants do not survive inside macrophages [37]. PhoP/Q is a two component transcriptional regulator that regulates expression of many *Salmonella* genes, including genes that modify LPS and make the bacteria more resistant to antimicrobial peptides [38]. A *phoP* mutant was nearly avirulent in our model (Figure 4). The mutant infected the cecum and caused localized inflammation, increased fecal water content, and mild diarrhea by day 2 after infection (Figure 4), as might be expected since the *phoP* mutation may even enhance epithelial cell invasion in vitro [39]. However, BALB/c.D2 mice rapidly reduced the numbers of *Salmonella* over the next week. The rapid clearance of *phoP*- bacteria from the colon was reflected in markedly reduced inflammation in the colon; on days 8/ 9 after infection there was no evidence of diarrhea and no inflammation in the cecum (Figure 4E). Thus, *phoP* mutant *Salmonella* could invade but could not survive in the colon, and they did not cause ongoing inflammation or a high diarrhea score, and they caused only a transient increase in fecal water content. We conclude from this that epithelial cell invasion by *Salmonella* is not sufficient to produce diarrhea in this model, though it may transiently impair epithelial cell function enough to decrease net water absorption.

PhoP/Q regulates many genes and it is not clear which of them are necessary for virulence. Among the genes that are indirectly regulated by PhoP/Q are *pmrA/B* [40], another two component regulatory system that controls a subset of the genes (*pmrF* operon) that *S. enterica* use to modify the negative charge on their LPS to increase resistance to antimicrobial peptides such as polymyxin B [41]. Therefore, we tested *pmrA* and *pmrF* mutants for their ability to cause colitis, and they were fully virulent. Thus, the profound attenuation of a *phoP/Q* mutant in the colitis model is not due to lack of expression of any of the *pmrA-*coregulated genes. PmrA/B control only a subset of the genes that regulate modifications of the LPS, and *pmrA* mutants remain resistant to some antimicrobial peptides such as cathelicidin. *phoP* mutants are more susceptible to cathelicidins, which may explain why *phoP* mutants are avirulent in this model [42].

The above results suggest that inflammation was connected with diarrhea. If inflammation is somehow responsible for diarrhea in *Salmonella* gastroenteritis, we reasoned that inflammatory mediators made by the host may contribute to diarrhea. We assessed the contribution of one host factor, reactive oxygen species (ROS), by infecting gp91^phox^ -deficient mice, which cannot generate ROS. Since the gp91^phox^ mutation is on a B6 genetic background, and gp91^phox^ is an X-linked gene, we were able to breed gp91^phox^ -/- mice on an ItyR genetic background by crossing homozygous female B6.gp91^phox^ -/- mice with male TF6 (Nramp1^G169^ transgenics). We then compared male F1 mice that had CGD with female littermates, who were carriers of the mutation and did not have CGD because of the Lyon effect. We found that male mice with CGD developed severe diarrhea when infected with 14028s, as severe as, or possibly worse than the controls. These results show that ROS from the host are not necessary to produce diarrhea. Not surprisingly, the male mice with CGD were also much more susceptible to *Salmonella* infection than were their sisters. These results extend the observations of Shiloh et al who reported that B6 mice with CGD are more susceptible to systemic *Salmonella* infections [43]. We also used the ItyR gp91^phox^ -/- mice to determine whether CGD mice were susceptible to oral infection with the *phoP* mutant, and they were not. This result implies that ROS are not required to kill *phoP* mutant *Salmonella* in vivo, and that there are non-oxidative killing mechanisms in mice that are an effective defense against *phoP* mutants but not WT *Salmonella*.

While we were doing these experiments Stecher et al reported that 2 strains of genetically resistant mice(ItyR) developed a severe form of *Salmonella* colitis, though the analysis presented was only of the cecal pathology [28]. In their experiments DBA/2 mice died of a progressive systemic infection, but the BALB/c.D2^Nramp1^ congenic mice that we studied did not die of infection. We are still uncertain why BALB/c.D2^Nramp1^ congenic mice died, but they probably died from colitis and not from overwhelming systemic *Salmonella* infection. In addition to Nramp1, there are a number of polymorphic genes in mice that influence resistance to systemic *Salmonella* infections [44], and these could explain the difference between the course of infection in BALB/c.D2^Nramp1^ congenic mice and the strains studied by Stecher et al.

In summary, we have developed a model of *Salmonella* colitis that mimics a severe form of human *Salmonella* gastroenteritis that is manifested as dysentery [45],[46],[47]. The infected mice showed several features of diarrhea and can be used to study the pathogenesis of diarrhea in mice. We found that neither SPI-1 mutant *Salmonella* that are unable to invade epithelial cells, nor *phoP* mutants that cannot survive in macrophages are able to cause diarrhea, and they caused much less inflammation in the colon than do wild type *Salmonella*. Diarrhea occurred even in the absence of the host's oxidative burst.

## Methods

### Mice

C57BL/6 (B6) and B6.gp91^phox^ -/- mice were purchased from Jackson Laboratory (Bar Harbor, MA). B6. Nramp1^G169^ transgenic mice (TF6) [48] and BALB/c.D2^Nramp1^ congenic mice [49] were bred at our institution under SPF conditions. In most experiments we included mice of both sexes and matched them for sex in all groups. We crossed female B6.gp91^phox^ -/- with male B6. Nramp1 transgenic mice in order to breed F1 males that carried the mutant B6.gp91^phox^ allele, and therefore have chronic granulomatous disease (CGD) on a Nramp1^G169^ genetic background, since the WT version of *Nramp1* is dominant. The female F1 mice are carriers of the B6.gp91^phox^ mutation and served as the controls for the male mice.

### Infection

We administered oral kanamycin (40mg) in 0.1 cc of saline by gavage. The following morning the mice fasted for 4 hours before we infected them by gavage with ∼5×10^3^ CFU of *S.* Typhimurium 14028s in 0.1 ml of 0.1M NaHCO_3_. Mice were allowed free access to food and water. They were weighed daily in some experiments. At necropsy we exposed the abdominal cavity and scored the severity of the diarrhea before we disturbed the tissues. We processed the tissues for culture as previously described [50], except for the ceca and feces. If there were formed feces in the distal colon, or if the mouse passed a formed stool when being removed from the cage, that was weighed and then homogenized in sterile saline and then diluted in saline for quantitative culturing on Hektoen Enteric (HE) agar with 20 µg/mL of kanamycin added. Each cecum was bisected and half used for culture after rinsing out the contents and then soaking the tissue in gentamicin 20mg/L for 30 minutes at room temperature. We then rinsed away the gentamicin with iced saline prior to grinding the tissue. All the tissues were homogenized mechanically in 1 ml of saline. The tissue slurry was serially diluted in sterile saline, and 100 µL of appropriate dilutions were cultured in duplicate on trypticase soy agar plates (spleens and mesenteric lymph nodes) or HE agar with 20 µg/mL of kanamycin added. All lactose-negative H_2_S positive colonies that grew on the HE-kanamycin plates were assumed to be *Salmonella*. Three cm long segments of the colon were processed similarly before they were cultured. If mice died before the time of sacrifice we assigned them the same number of CFU that we recovered from the most intensely infected living mouse, a conservative estimate. If we could not obtain feces from a mouse because of diarrhea we did not include that mouse in our calculations. Feces were weighed before we processed them and we calculated CFU/gram of feces.

The scoring system that we used to assess the severity of the diarrhea is shown in Table 1. To determine the water content of the feces we removed all the luminal contents from the distal 3 cm of the colon and placed them into Eppendorf tubes. The tubes with the lids off were weighed and then dried in a SpeedVac (Thermo Savant, Waltham, MA) for 4-6 hours until all samples were dry. The tubes were reweighed and the percentage decrease in weight was calculated. This method is a modification of the one used by Gutman et al. to measure water content of feces of *C. rodentium*-infected mice [25].

### Bacteria

We constructed derivatives of Typhimurium strain 14028s containing a kanamycin resistance cassette (kanR) inserted in a genetically silent region downstream from the *spv* operon in the virulence plasmid. This *S. enterica* serovar Typhimurium 14028s KanR strain [51] was used to allow the bacteria to survive in the colon of mice treated with oral kanamycin. *S.* Typhimurium 14028s *invA::aphT* has been described previously [52]. The *phoP*::Tn*10* allele [19] was transduced into the 14028s KanR strain using phage P22, as described [51]. *pmrA* [41] and *pmrF* [53] mutants were transduced into the 14028s KanR strain as above. Bacteria were grown overnight in TSB, and washed and resuspended as previously described [54].

### Histology

We filled the entire colon with Bouin's solution, tightly coiled the colon around a wooden applicator stick, and immersed the coiled colon in Bouin's solution. After embedding in paraffin we made 5 mm thick sections that we stained with hematoxylin and eosin (H&E). Each slide was coded and read blind by the PI.

### Statistics

Results were analyzed using GraphPad Prism version 4 (San Diego, CA). Multiple groups were compared using a one way ANOVA and the differences between two groups were examined with the Kruskal-Wallis statistic. To compare two groups of results we used the non-parametric Mann-Whitney U test. P values of <0.05 were considered significant.
